# 21st Century Sea Ice Loss Will Upend 11,700 Years of Stable Habitat for Bowhead Whales

**DOI:** 10.1002/ece3.71377

**Published:** 2025-05-20

**Authors:** Nicholas A. Freymueller, Eline D. Lorenzen, Stuart C. Brown, Carsten Rahbek, Damien A. Fordham

**Affiliations:** ^1^ Environment Institute and School of Biological Sciences University of Adelaide Adelaide South Australia Australia; ^2^ Center for Macroecology Evolution and Climate Globe Institute, University of Copenhagen Copenhagen Denmark; ^3^ Globe Institute University of Copenhagen Copenhagen Denmark; ^4^ Center for Global Mountain Biodiversity, Globe Institute University of Copenhagen Copenhagen Denmark; ^5^ Danish Institute for Advanced Study University of Southern Denmark Odense Denmark; ^6^ Institute of Ecology Peking Institute Beijing China

## Abstract

Bowhead whales ( *Balaena mysticetus* ) are strongly associated with Arctic sea ice during their crucial summer feeding period. However, anthropogenic climate change is causing a decline in sea ice concentrations, threatening bowhead whale suitable habitat. To characterise the long‐term affinity of bowhead whales to sea ice across the Holocene and project the response of populations to 21st century climate change, we built ecological models of occurrence–environmental relationships using distribution‐wide fossil, historical, and contemporary records. We found that throughout the Holocene, bowhead whale habitat suitability was consistently highest in summer average sea ice concentrations of 15%–30%. Projecting these models forward in time to 2100 ce showed that 21st century climate change is set to erode these critical sea ice conditions, resulting in the circumpolar range of bowhead whales contracting by up to 75%. We project that during this century, habitat suitability will decline in all four management populations of bowhead whales by at least 52%, with suitable habitat predicted to vanish completely in the Sea of Okhotsk. It is likely that most viable habitat for bowhead whales will exist outside their current distribution by the end of the century, directly impacting conservation policies. Our results further highlight the vulnerability of Arctic marine endemics in a warming world, showcasing how knowledge of the past can strengthen predictions of species future vulnerability to rapid ocean warming.

## Graphical abstract

We built ecological niche models for bowhead whales using fossil, historical, and contemporary occurrence and environmental data. We found that the suitability of bowhead whale habitats was stable over the Holocene, with with whales preferring environments with 15%–30% sea ice cover. However, future projected loss of Arctic sea ice will upend this relationship, threatening bowhead whales.

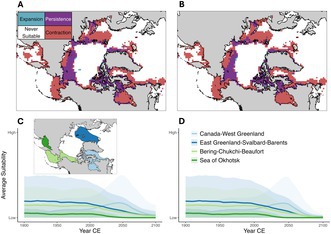

## 1 Introduction

Arctic marine ecosystems are currently experiencing rapid restructuring due to anthropogenic global warming (Wassmann 2011). Decreases in Arctic sea ice and earlier onsets of sea ice melting during summer (Karami et al. 2023) are disrupting species interactions (Alabia et al. 2023), leading to changes in ecosystem functioning (Wassmann 2011). Warming sea temperatures are enabling sub‐Arctic species to expand their distributions poleward (Kortsch et al. 2015), amplifying competition with Arctic endemics (Alabia et al. 2023). Many climate modelling projections indicate that summers in the Arctic Ocean will be ice free by 2100 ce (Boé et al. 2009). Thus, Arctic marine ecosystems will continue to experience rapid changes in the coming decades.

Marine megafaunal species that are endemic to the Arctic tend to rely directly on, or are strongly associated with, specific sea ice conditions for their persistence (Laidre et al. 2008). Thus, sea ice retreat will likely cause population declines resulting in extirpations and changes in geographic distributions (Insley et al. 2021; Ragen et al. 2008). This includes bowhead whales ( *Balaena mysticetus* ), the only baleen whale found in the Arctic year‐round. Bowhead whales consume the majority of their annual calories in highly productive summer foraging areas on shallow (< 200 m) areas of continental shelves (Matthews and Ferguson 2015). Here, they structure ecosystems during summer by consuming vast amounts of zooplankton (Moore and Stabeno 2015), facilitating nutrient cycling throughout the Arctic (Laidre et al. 2007). Satellite telemetry indicates that bowhead whales have a particular affinity for Arctic summer sea ice, generally occurring in shallow coastal environments containing ≥ 15% sea ice concentration (Ferguson et al. 2010; Kovacs et al. 2020). Therefore, future loss of critical summer habitat is likely to negatively affect their long‐term survival.

Bowhead whales are currently classified as ‘Vulnerable’ by the IUCN (Cooke and Reeves 2018) following a population bottleneck caused by commercial whaling from ~1530 to 1910 ce (Thewissen and George 2021), where they were extirpated from several parts of their historic range (Baird and Bickham 2021). Current abundances are estimated at ~25% of their pre‐whaling size, and recovery patterns have varied across the four managed populations (termed stocks; Figure S1) (Baird and Bickham 2021). The Bering‐Chukchi‐Beaufort and Canada‐West Greenland stocks show marked signs of recovery, whereas the East Greenland‐Svalbard‐Barents and Sea of Okhotsk stocks remain heavily depleted (Thewissen and George 2021).

Investigations of the vulnerability of bowhead whales to future climate change have used ecological niche modelling (ENM) approaches to determine potential shifts in habitat suitability, finding that their future summer distribution will both contract and shift northward (Chambault et al. 2022; Foote et al. 2013). However, these previous studies generated ENMs using only contemporary distribution data from partial areas of the circumpolar range of the species. No forecasts of climate exposure and vulnerability of bowhead whales have yet utilized records from the Sea of Okhotsk, despite whaling records indicating this region was once broadly populated by bowhead whales (Townsend 1935). Moreover, no future projections have leveraged past distribution data, despite historical whaling having truncated the current distribution of bowhead whales (Baird and Bickham 2021). Thus, in the absence of range‐wide and past distribution data, existing forecasts of habitat suitability are likely to underestimate the full range of ecological conditions that are habitable to bowheads, potentially mischaracterizing future suitability patterns (Faurby and Araújo 2018).

Radiocarbon‐dated bowhead whale fossils and historical archives, such as commercial whaling logbooks, provide new opportunities to incorporate past distribution patterns into ENMs, potentially improving projections of bowhead whale vulnerability to climate change through better knowledge of occurrence‐habitat relationships (Fordham et al. 2020). Integration of these data into ENMs provides a long‐term baseline of habitat suitability that is unaltered by the anthropogenic effects of commercial whaling, allowing for more precise characterisation of potential future threats (Nogués‐Bravo 2009).

Here, we reconstruct a baseline of bowhead whale habitat suitability across the Holocene (11,700 years BP–2020 ce) and estimate the long‐term association of bowhead whales with summer sea ice concentration. To this end, we integrated bowhead whale occurrences from: (i) the Holocene fossil record; (ii) historical commercial whaling archives; and (iii) contemporary occurrence records spanning the entire circumpolar range of bowhead whales. We combined occurrence data with paleo‐environmental reconstructions from climate models using a multi‐temporal ENM framework (Nogués‐Bravo 2009). To understand how future climate warming scenarios will alter bowhead whale summer distributions, we projected these models to 2100 ce under two contrasting Shared Socioeconomic Pathways (SSP 2–4.5 and SSP 5–8.5) (O'Neill et al. 2016). We show that while habitat suitability was largely stable throughout the Holocene, it is projected to decline markedly in the coming decades.

## 2 Methods

### 2.1 Occurrence Data

We accessed bowhead whale occurrence data from a variety of sources spanning the past 11,700 years. These were: (i) 823 geolocated and radiocarbon‐dated, marine reservoir‐corrected bowhead fossil (pre‐1500 ce) occurrences (pre‐1500 ce), which we sourced from Westbury et al. (2024); (ii) 3374 historical (~1500–1910 ce) occurrence records from European and North American commercial whaling logbooks that document where whalers saw or harvested whales; and (iii) 37,028 contemporary (post 1910 ce) occurrence records from biodiversity databases and primary literature (Freymueller et al. 2023). These data span the entire circumpolar and sub‐Arctic range of bowhead whales, encapsulating bowhead occurrences from the distant and recent past when climatic conditions were different to those today (Table S1). We filtered historical and contemporary occurrences to summer months (June–October). We used all fossil occurrence records, assuming that they represented summer sea ice‐entrapments (Dyke et al. 1996; Westbury et al. 2024). We retained 1308 occurrences for use in modelling after data‐cleaning and spatially thinning records to one occurrence per 90 × 90 km grid cell per 35‐year time bin, with the final bin ending in 2020 ce (Aiello‐Lammens et al. 2015; Feng et al. 2019). We used a 35‐year time bin because it covers the bowhead whale generation length (Pacifici et al. 2013). This spatial and temporal scale is appropriate for using multi‐temporal ENMs to estimate habitat suitability for a wide‐ranging, highly mobile species, such as the bowhead whale (Westbury et al. 2024). It also addresses issues with reconstructing fine‐scale climate variability (Fordham et al. 2024). Because calibrated ages of fossils spanned multiple 35‐year bins, we generated pseudo‐replicates of fossil occurrences in each bin, then weighted them according to their 95% calibrated interval such that the total weight for a single fossil occurrence summed to 1.

We partitioned our occurrence data into ten folds for cross‐validation (Peterson et al. 2011). We stratified folds by stock and type of record (i.e., fossil, historical, contemporary). We opted for this cross‐validation approach because commercial harvests truncated the geographic distribution of the bowhead whale (Baird and Bickham 2021), meaning model testing on contemporary records alone would be biased.

### 2.2 Climate and Environmental Data

Holocene climatic/environmental data came from a HadCM3B‐M2.1 coupled atmosphere–ocean general circulation model (Armstrong et al. 2019). Specifically, we extracted three summer‐averaged (June–October) variables—sea ice concentration (SIC), sea surface temperature (SST), and sea surface salinity (SSS)—that are important for the ecology and demography of bowhead whales, and which have previously been used to construct ecological niche models for the species (Chambault et al. 2022; Westbury et al. 2024).

Holocene bathymetry came from the ICE‐5G isostatic model (Peltier 2004) which is an input to the HadCM3B‐M2.1 model. Bathymetry was used to capture bowhead whale affinity with shallow coastal environments during summer (Matthews et al. 2020). We did not use measures of net primary productivity as they were unavailable prior to 1850 ce, and their future estimates are highly uncertain (Vancoppenolle et al. 2013). We bilinearly interpolated bathymetry from its native 500‐year intervals to 35‐year intervals and used contemporary bathymetry for future projections. We regridded our data to 90 × 90 km to avoid producing downscaling artefacts in the paleoclimate data (Beyer et al. 2020).

We used a five‐model weighted ensemble from the Coupled Model Intercomparison Project Phase 6 (CMIP6) to generate contemporary and future climate projections. The CMIP6 ensemble spanned 1850–2100 ce and included the following models: ACCESS‐ESM1‐5; MIROC6; MPI‐ESM1‐2‐HR; MPI‐ESM1‐2‐LR; and MRI‐ESM2‐0. Models were selected and weighted based on their ability to recreate sea ice dynamics in the Arctic (Long et al. 2021). Future climate simulations (2021–2100 ce) of SIC, SST, and SSS were based on Shared Socioeconomic Pathways (SSPs), which the Intergovernmental Panel on Climate Change uses to project future climate based on government climate policies (O'Neill et al. 2016). We investigated two different SSP scenarios: a ‘middle of the road’ scenario (SSP 2–4.5), and a ‘fossil‐fuelled development’ scenario (SSP 5–8.5) (O'Neill et al. 2016). These SSP scenario are commonly used as best‐ and worst‐case scenarios in assessments of future climate change impacts on marine biodiversity (Mellin et al. 2024). Their comparison allowed us to bracket future habitat suitability changes for bowhead whales.

We bias‐corrected CMIP6 climate data using the HadCM3B‐M2.1 data over the interval 1850–1950 ce. We used an additive delta method for SST and a multiplicative delta method for SSS (Armstrong et al. 2019). Delta methods are not suitable for correcting SIC as there are natural bounds at 0% and 100%. Furthermore, there are likely to be non‐linear relationships between climate and environmental drivers of sea ice extent which cannot be accounted for via delta methods (Beyer et al. 2020). Therefore, we used a beta‐regression Generalised Additive Model (GAM) (Wood 2017) to model non‐linear relationships between SIC and our climate/environmental drivers, and ensured that predicted (i.e., corrected) SIC was measured as percent change. Our beta‐GAM estimated CMIP6‐derived SIC (bound between 0% and 100%) as a function of: HadCM3B‐M2.1‐derived SIC; bathymetry; distance from coastline; month; and a tensor product gaussian process for longitude and latitude.

All environmental data were averaged across 35‐year intervals and regridded to a 90 × 90 km spatial resolution for oceanic areas above 45° N using a distance‐weighted interpolation method implemented in Climate Data Operators software (Schulzweida 2023).

### 2.3 Ecological Niche Model

We matched occurrence records in time and space to the four climate and environmental variables: SIC, SST, SSS, bathymetry. Records that fell on land, owing to the coarse scale of the land‐sea mask (1° × 1° grid cells), were shifted to the nearest oceanic grid cell if < 170 km away. We calculated the environmental conditions of each occurrence using the average of the grid cell and its eight nearest grid cells. This mitigated biases from climate data processing and bowhead whale movements over the summer (Chambault et al. 2022; Kovacs et al. 2020).

Initial data visualisation revealed that contemporary occurrence records for bowhead whales exist only in a subset of the environmental conditions that the species has persisted in throughout the Holocene (Figure S2), indicating that their niche has been truncated. This supports the need for a multi‐temporal ecological niche model (ENM) framework for projecting future changes in habitat suitability.

We modelled the potential realised niche of bowhead whales over the past 11,700 years using a 4‐dimensional (SIC, SST, SSS, bathymetry) ‘gaussian’ hypervolume ENM with the hypervolume R package (Blonder et al. 2014). This presence‐only method is suitable for incorporating historical and fossil occurrences into ENMs using multi‐temporal methods (Myers et al. 2015). Furthermore, ENMs built with the hypervolume approach do not extrapolate beyond those used to train the model (Blonder et al. 2014). Thus, our projections are less likely to generate false positives of suitable habitat under novel environmental conditions (Blonder et al. 2014). Models were tuned by varying hyperparameters of the kernel bandwidths (0.5–2.5 in steps of 0.25), number of standard deviations (3:5), and probability thresholds (0.95, 0.975, 0.99). Model projections were further tuned by varying ranges for edges.zero‐distance.factor (0.5–5 in steps of 0.5) and weight.exponent (−1:−4).

We calculated ROC‐AUC values (using randomly‐generated points within the hypervolume as pseudo‐background data), Continuous Boyce Index, and Sensitivity using ten‐fold cross‐validation for each tuning iteration. We standardised these validation metrics, summed them together, and selected the ‘best’ model tuning based on the ‘one‐standard error’ rule (Burnham and Anderson 2002). Thus, the ‘best’ model chosen performed well across multiple commonly used ENM validation metrics (Peterson et al. 2011): AUC = 0.71 ± 0.02; Boyce = 0.90 ± 0.05; Sensitivity = 0.85 ± 0.01.

### 2.4 Projections Under Climate Change

We used our multi‐temporal ENM to project habitat suitability throughout the Holocene and to forecast suitability into the future under both SSP scenarios, retaining values above the 10th percentile of habitat suitability threshold (Peterson et al. 2011). We did this using projected changes in SIC, SST and SSS for the period between 2021 and 2100 ce. We aggregated grid cells into sea ice isobands of 0%–15%, 15%–30%, 30%–45% and > 45%. We quantified average habitat suitability within each isoband and calculated the proportion of suitable grid cells within each isoband, for the Holocene (prior to 1850 ce) and since the start of the industrial revolution into the future (1850–2100 ce) across the entire distribution of the bowhead whale. Additionally, we compared modelled projections with known summer congregation sites for bowhead whales in the Bering‐Chukchi‐Beaufort stock (Baird and Bickham 2021; Citta et al. 2018; Druckenmiller et al. 2018; Harwood et al. 2017), showing that our ENM can distinguish between known optimal and suboptimal areas of bowhead whale distribution (Figure S3).

## 3 Results

**FIGURE 1:**
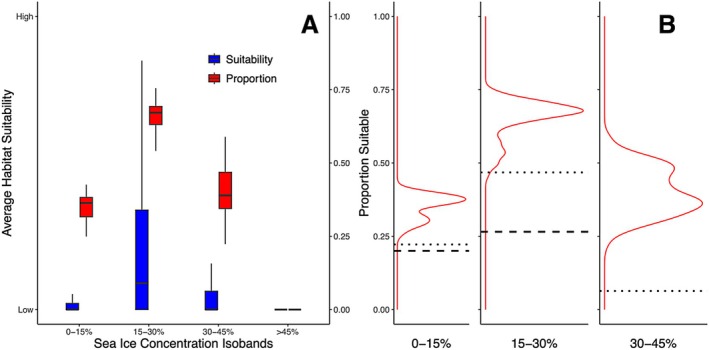
Habitat suitability and its relationship to sea ice concentration for bowhead whales. (A) Average habitat suitability (blue) and average proportional area suitable for bowhead whales (red) in four isobands of sea ice concentration (0%–15%; 15%–30%; 30%–45%; > 45%) for the Holocene (11,700 BP—1850 ce). (B) Frequency distributions of proportion‐of‐area‐suitable in three sea ice isobands (0‐15%; 15‐30%; 30‐45%) for the Holocene prior to 1850 ce and for 2100 ce under two different shared socioeconomic pathways (SSPs): Moderate (SSP 2‐4.5, dotted black line); and High (SSP 5‐8.5, dashed black line). Of note, Arctic sea ice concentration does not exceed 30% by 2100 ce under SSP 5–8.5.

Our multi‐temporal ENM showed that environments with average summer sea ice concentration (SIC) between 15% and 30% contained the highest average bowhead whale habitat suitability (Figure 1A; Video S1). These areas also provided the largest proportion of habitable area throughout the Holocene (Figure 1A–1B).

**FIGURE 2:**
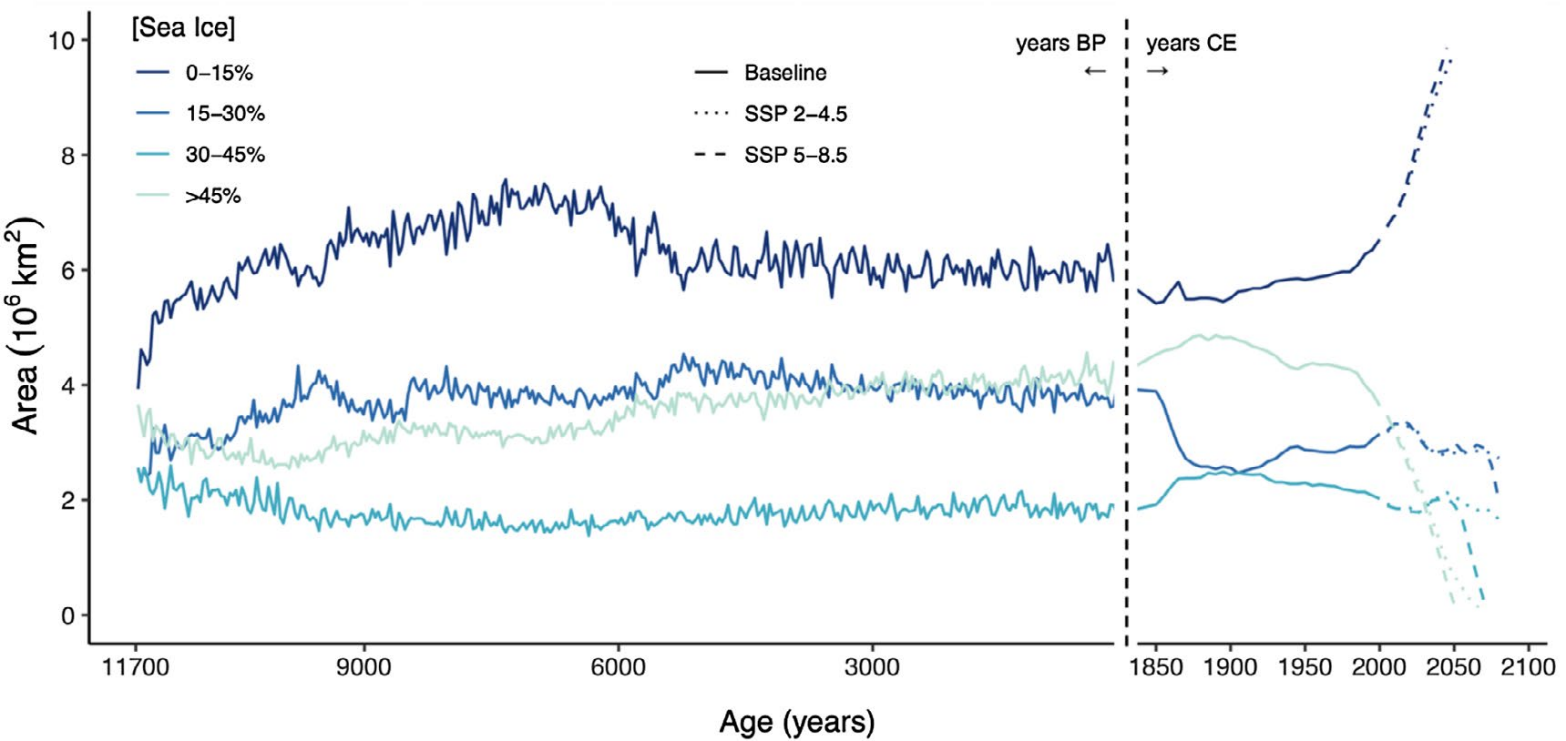
Spatial extent of past and future Arctic sea ice concentration. Projected extent (10^6^ km^2^) of sea ice concentration (SIC) in four isobands (0%–15%; 15%–30%; 30%–45%; > 45%, indicated by different shades of blue) from 11,700 years BP—2100 ce. Solid lines show area projections before 2020 ce and broken lines for the future. Future projections are for moderate (2–4.5; dotted line) and high (5–8.5; dashed line) SSP scenarios.

Although the spatial extent of sea ice isobands has remained relatively constant over the Holocene, they are forecast to change substantially, with large 21st century decreases projected for all isobands with sea ice > 15%, regardless of SSP scenario (Figure 2, Videos S2 and S3). Our modelling shows that this decrease in sea ice will negatively impact the area of occupiable habitat for bowhead whales, particularly in the most favoured 15%–30% isoband (Figure 1B). We project that under a SSP 2–4.5 scenario, the area of suitable habitat in the 15%–30% isoband will decrease by ~50% by 2100 ce, compared to a Holocene baseline (Figure 1B). Under a more severe SSP 5–8.5 scenario, our analysis indicates even larger decreases, with ~90% decline in the area of suitable habitat in the 15%–30% isoband by 2100 ce (Figure 1B).

**FIGURE 3:**
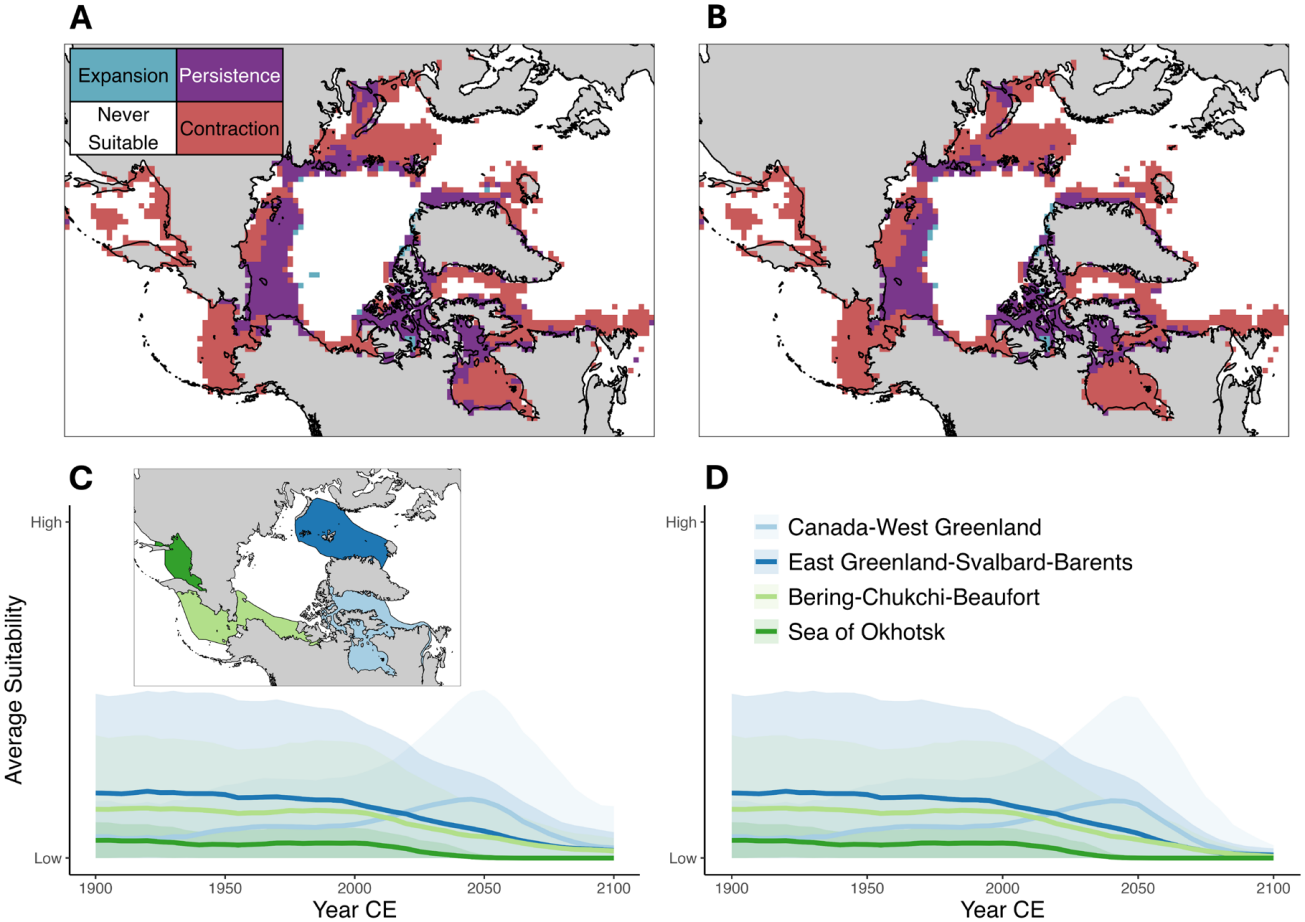
Projected changes of bowhead whale suitable habitat under anthropogenic climate change. Top panels show projected expansion, contraction, and persistence of occupiable habitat in 2100 ce, under two shared socioeconomic pathways (SSPs): (A) SSP 2–4.5 and (B) SSP 5–8.5. Change is calculated from a long‐term Holocene baseline (11,700 BP—1850 ce). Lower panels show climate‐driven change in average habitat suitability since 1900 ce for bowhead whales in the four different management stocks under (C) SSP 2–4.5 and (D) SSP 5–8.5 scenarios. Whale stocks are: Canada‐West Greenland (light blue); East Greenland–Svalbard–Barents (dark blue); Bering–Chukchi–Beaufort (light green); Sea of Okhotsk (dark green). Shaded areas show ±1 standard deviation.

Our modelling of past, present and future patterns of habitat suitability shows that the distribution of bowhead whales is expected to contract and shift markedly north during the 21st century, regardless of SSP scenario (Figure 3A–D). We show that patterns of habitat suitability were largely stable during the Holocene prior to commercial whaling, being broadly consistent with the contemporary distribution of bowhead whales (Figure S4). However, we predict that these patterns will change dramatically by 2100 ce, with the extent of potentially suitable habitat decreasing by 64%–75% across the species' global distribution, depending on SSP. In areas where the suitable habitat of bowhead whales is predicted to persist, habitat quality (predicted as ENM suitability) is almost always lower, most notably in areas where bowhead whales currently congregate during the summer (Figures S3 and S5). While our models do project some areas where bowhead whale occupiable habitat will expand, these areas are very small compared to areas that will vanish (Figure 3A,B).

We project that the suitability of viable habitat within the current boundaries of all four recognised bowhead whale stocks will decrease by 52%–100% by 2100 ce under a SSP 2–4.5 scenario and will decrease by 90%–100% under a SSP 5–8.5 scenario (Figure 3C,D; Table S2). Average suitability in the Pacific stocks (Bering‐Chukchi‐Beaufort and Sea of Okhotsk) is expected to experience the greatest declines. Our models predict that the current Sea of Okhotsk stock area, which is already at the warmest edge of the bowhead ecological niche (Figure S6), will be uninhabitable by ~2060 ce (Figure 3C,D).

## 4 Discussion

Our modelling of 11,700 years of bowhead whale occurrence and environmental archives indicates that the most suitable habitat conditions in summer for bowhead whales are in environments with 15%–30% sea ice concentration. We find that the spatial extent of these conditions remained relatively unchanged for the entire Holocene, resulting in a broadly stable potential distribution of bowhead whales. However, the affinity of bowhead whale habitat with sea ice concentrations is predicted to change sharply by the end of the 21st century, regardless of the climate warming scenario. Declines in sea ice concentrations across the Arctic are likely to cause both contraction and northward shifts in the distribution of bowhead whales, with likely reductions in population abundance (VanDerWal et al. 2009). We project that within contemporary stock boundaries, suitable habitat for bowhead whales will decline by 64%–75% by 2100 ce, with stocks in the Pacific Ocean set to experience the largest losses, making bowhead whales highly vulnerable to near‐future anthropogenic climate change.

We project that by 2100 ce, most suitable bowhead whale habitat will be outside the current distribution of the species. In the Sea of Okhotsk, suitable habitat is projected to vanish entirely by ~2060 ce, casting doubt on whether bowhead whales will be able to persist here. Further, our estimates of rates of habitat loss may be conservative, as the current suite of CMIP6 models (used in this study) tend to underpredict decadal rates of summer sea ice loss (Shen et al. 2021).

We expect that bowhead whales will not track northward shifts in habitat suitability beyond the continental shelf, even if such areas contain sea ice concentrations of 15%–30% at the end of this century. This is because deep environments in the central Arctic are unlikely to sustain sufficient primary productivity to support food resources for bowhead whales (Vancoppenolle et al. 2013). Although climate models disagree on whether Arctic Ocean surface net primary productivity will increase or decrease by 2100 ce, these models agree that the central Arctic Ocean will remain highly unproductive even if it becomes ice‐free (Vancoppenolle et al. 2013).

Verification tests, done in addition to cross‐validation, show that our modelling does well at distinguishing between optimal and suboptimal contemporary habitats for bowhead whales (SI Figure 3), providing additional confidence in our projection that remaining habitats for bowhead whales in 2100 ce will be of relatively low quality. The decline and loss of suitable habitat in these areas can be expected to deplete population abundances of bowhead whales, as habitat suitability and abundance are generally positively correlated (de la Fuente et al. 2021).

Our study indicates that contemporary bowhead whales are generally found in similar average summer environmental conditions across their distribution, except in the Sea of Okhotsk (Figures S2 and S6). Today, environments in the Sea of Okhotsk represent low quality habitat for bowhead whales and are characterised by warm summer sea surface temperature (10°C–13°C) and low (≤ 10%) summer SIC. These environments are already at the warmest edge of the niche of bowhead whales, suggesting populations in this stock are the most vulnerable to anthropogenic ocean warming and associated SIC loss. If bowhead whales are indeed as sensitive to rising sea surface temperatures and low summer sea ice concentration as we project, the bowhead whales in the Sea of Okhotsk are at severe risk of extirpation in the coming decades, particularly as they appear unable to migrate to other stocks by moving northeast around Kamchatka (Ivashchenko and Clapham 2010).

It is possible that projected declines in habitat suitability for the bowhead whales in the Sea of Okhotsk could, to an extent, reflect a reduced understanding of marginal environmental conditions that bowhead whales can persist in. Even though our study is the first to use occurrence data from the Sea of Okhotsk to build ENMs, only ~6% of all occurrences came from this stock. This is because there are no bowhead whale fossils from this area, relatively few contemporary records, and relevant whaling data mainly come from a short historical period in the 1850s and 1860s. However, by incorporating all available ancient and historic occurrence data into our ENMs, we largely avoided the truncating effect of commercial whaling on habitat suitability, providing arguably better estimates of the effects of climate change on habitat suitability for bowhead whales across all four stocks (Faurby and Araújo 2018).

Fossils from the late Pleistocene show that bowhead whales were once distributed to the south of the current‐day stock boundaries, including southern Scandinavia and the paleo‐Champlain Sea in southern Ontario, Canada (Aaris‐Sørensen et al. 2010; Harington 1981). Our modelling indicates that these areas of their former range have been uninhabitable since at least the beginning of the Holocene (Video S1). Thus, our model is consistent with the fossil record from these areas denoting extirpation by the Early Holocene. The first evidence of human hunting of bowhead whales does not occur until the Late Holocene in Greenland (Seersholm et al. 2016). Thus, bowhead whales likely became extirpated from these areas due to climatic warming at the end of the Pleistocene. Since past warming has probably truncated the warmest edges of the bowhead whale geographic range, these observations suggest that future climatic warming and loss of summer habitat for bowhead whales is likely to be detrimental.

Contemporary bowhead whale populations are already responding to Arctic warming by spending more time in ice‐free conditions in the summer (Citta et al. 2018; Druckenmiller et al. 2018; Harwood et al. 2017). However, commercial whaling logbooks show that this is a recent phenomenon. Commercial whalers regularly reported bowhead whales in sea ice in southern areas of their distribution (i.e., areas south of the Bering Strait) in late summer during and before the early 1900s (Figure S7) (Bockstoce and Botkin 1983; Mahoney et al. 2011; Walsh et al. 2019). Our modeling suggests that this distributional response to a warming climate will most likely intensify in coming decades, underscoring the need for a long‐term understanding of habitat use by bowhead whales.

Our spatially explicit predictions of past and future habitat suitability will be useful for management and conservation agencies seeking to safeguard bowhead whales (Guisan et al. 2013). By identifying the extent and location of bowhead whale habitat that is likely to be lost in coming decades in response to human‐induced climate change, and what areas may provide new habitat, our projections provide vital information to guide future management efforts. More broadly, our projections can be integrated with spatially explicit population models to directly quantify the likely risk of population extirpation from climate change, and to test different management intervention strategies (Fordham et al. 2013).

The decline in suitable habitat for bowhead whales that we predict for this century is unprecedented in the last 11,700 years of their eco‐evolutionary history and will likely lead to widescale local and regional extirpations. Our findings indicate extreme decreases in habitat suitability, even when considering a long‐term perspective. This highlights the severity of future climate change in the Arctic and cautions the heightened vulnerability of this keystone Arctic endemic species.

## Author Contributions

**Nicholas A. Freymueller:** conceptualization (lead), data curation (lead), formal analysis (lead), methodology (lead), visualization (lead), writing – original draft (lead). **Eline D. Lorenzen:** conceptualization (supporting), funding acquisition (equal), writing – original draft (supporting), writing – review and editing (equal). **Stuart C. Brown:** conceptualization (equal), formal analysis (supporting), supervision (supporting), visualization (supporting), writing – review and editing (equal). **Carsten Rahbek:** conceptualization (supporting), writing – review and editing (supporting). **Damien A. Fordham:** conceptualization (equal), formal analysis (supporting), funding acquisition (equal), methodology (supporting), supervision (equal), visualization (supporting), writing – review and editing (equal).

## Conflicts of Interest

The authors declare no conflicts of interest.

## Supporting information


**Data S1.** **Figure S1.** Distribution of the four bowhead whale management stocks. Whaling stocks are: East Greenland‐Svalbard‐Barents (dark blue); Canada‐West Greenland (light blue); Bering‐Chukchi‐ Beaufort (light green); Sea of Okhotsk (dark green).
**Figure S2.** Pairwise 2‐dimensional cross‐sections of the bowhead whale hypervolume. Colours represent post‐processed occurrence records coming from fossil (green), historical (orange), and contemporary (blue) time periods. Density plots show the distribution of occurrence records along three of the four variables we included in the bowhead whale niche model: sea ice concentration (SIC); sea‐surface temperature (SST); and sea‐surface salinity (SSS). Bathymetry is not shown. The pie chart shows the proportion of records coming from each time period.
**Figure S3.** Relationship between habitat suitability and important current‐day congregation sites for bowhead whales in the Bering‐Chukchi‐Beaufort stock. (A) summer‐averaged (June–October; 1900–2020 ce) habitat suitability patterns, with summer high‐congregation areas highlighted in blue along the Beaufort Sea coast. (B) Habitat suitability patterns (1900–2100 ce) under a Shared Socioeconomic Pathway (SSP) 2–4.5 climate scenario in core use (blue) and non‐core use areas (black). Dashed lines denote ±1 standard error. (C) Habitat suitability patterns (1900–2100 ce) under a SSP 5–8.5 climate scenario in core use (blue) and non‐core use areas (black). Dashed lines denote ±1 standard error. Our niche model positively identifies core congregation areas as having high habitat suitability. However, we project suitability in these core areas rapidly decline in the future regardless of SSP scenario, suggesting bowhead whales may cease being able to use them.
**Figure S4.** Spatial pattern of habitat suitability for the Holocene baseline (11,700 BP—1850 ce). Blue denotes the area where the 15%–30% sea ice concentration isoband has persisted for ≥ 50% of Holocene summers.
**Figure S5.** Three‐tiered congruence maps (no suitability, low suitability, high suitability) between the Holocene baseline (11,700 BP—1850 ce) and the two different Shared Socioeconomic Pathway (SSP) scenarios at 2100 ce (2–4.5 [A]; 5–8.5 [B]). Even in areas that will remain suitable by 2100 ce, suitability will remain low areas that still contain suitable habitat.
**Figure S6.** 3‐dimensional representation of the hypervolume of climatic conditions that bowhead whales occupy based on fossil, historical, and contemporary occurrence data. Different colours show different bowhead stocks (East Greenland‐Svalbard‐Barents [blue]; 2 = Canada‐West Greenland [green]; Bering‐Chukchi‐Beaufort [yellow]; 4 = Sea of Okhotsk [red]). Note that the occurrence relationship with bathymetry is not shown.
**Figure S7.** The latitude and day‐of‐year that historical Bering‐Chukchi‐Beaufort bowhead whale strikes were also observed on the same day as Arctic sea ice. The red line is an approximate latitude for the Bering Strait, indicating that bowhead whales were associated with summer sea ice south of the strait during the period of commercial harvest (1848–1914). Data are from Mahoney et al. (2011) based off original logbook extractions by Bockstoce and Botkin (1983).

**Data S2.** **Table S1.** Data sources and sample sizes of the bowhead whale occurrence data before and after data cleaning. For historical sources, citations marked with an asterisk were digitised in Smith et al. (2012). Some modern and historical data sources contain the same or redundant data, which were removed via spatial thinning (Aiello‐Lammens et al. 2015). The majority of modern occurrence records came from recent high‐resolution satellite telemetry data, which is why so much of it was removed via spatial thinning.
**Table S2.** Percent declines in suitable habitable area for bowhead whales in 2100 ce under SSP 2–4.5 and SSP 5–8.5 emissions scenarios. Declines are shown for each population as well as declines across the entire bowhead whale circumpolar range.

**Video S1.** Habitat suitability patterns for bowhead whales from 11,700 years before present to 2020 ce. Blue curves indicate where areas containing the favoured 15%–30% sea ice concentration conditions are present. Note that the land‐sea mask changes throughout the Holocene due to sea level rise following the melting of the Laurentide Ice Sheet.

**Video S2.** Habitat suitability patterns for bowhead whales from 1850 to 2020 ce under a ‘hopeful’ Shared Socioeconomic Pathway 2–4.5 scenario. Blue curves indicate where areas containing the favoured 15%–30% sea ice concentration conditions are present.

**Video S3.** Habitat suitability patterns for bowhead whales from 1850 to 2020 ce under a ‘fossil‐fuelled development’ Shared Socioeconomic Pathway 5–8.5 scenario. Blue curves indicate where areas containing the favoured 15%–30% sea ice concentration conditions are present.

## Data Availability

All modelling was done in R and supporting data/code can be accessed from the following link: https://figshare.com/projects/Code_and_data_for_modelling_11_700_years_of_stable_habitat_for_bowhead_whales/204249 (Freymueller et al. 2023).
