# Supplementary material for: 21st Century Sea Ice Loss Will Upend 11,700 Years of Stable Habitat for Bowhead Whales
Source: Ecol Evol. 2025 May 20;15(5):e71377. doi: 10.1002/ece3.71377 (PMC12092169; doi:10.1002/ece3.71377)
Supplement: Supplementary file 1 — Data S1. Figure S1. Distribution of the four bowhead whale management stocks. Whaling stocks are: East Greenland‐Svalbard‐Barents (dark blue); Canada‐West Greenland (light blue); Bering‐Chukchi‐ Beaufort (light green); Sea of Okhotsk (dark green). Figure S2. Pairwise 2‐dimensional cross‐sections of the bowhead whale hypervolume. Colours represent post‐processed occurrence records coming from fossil (green), historical (orange), and contemporary (blue) time periods. Density plots show the distribution of occurrence records along three of the four variables we included in the bowhead whale niche model: sea ice concentration (SIC); sea‐surface temperature (SST); and sea‐surface salinity (SSS). Bathymetry is not shown. The pie chart shows the proportion of records coming from each time period. Figure S3. Relationship between habitat suitability and important current‐day congregation sites for bowhead whales in the Bering‐Chukchi‐Beaufort stock. (A) summer‐averaged (June–October; 1900–2020 ce) habitat suitability patterns, with summer high‐congregation areas highlighted in blue along the Beaufort Sea coast. (B) Habitat suitability patterns (1900–2100 ce) under a Shared Socioeconomic Pathway (SSP) 2–4.5 climate scenario in core use (blue) and non‐core use areas (black). Dashed lines denote ±1 standard error. (C) Habitat suitability patterns (1900–2100 ce) under a SSP 5–8.5 climate scenario in core use (blue) and non‐core use areas (black). Dashed lines denote ±1 standard error. Our niche model positively identifies core congregation areas as having high habitat suitability. However, we project suitability in these core areas rapidly decline in the future regardless of SSP scenario, suggesting bowhead whales may cease being able to use them. Figure S4. Spatial pattern of habitat suitability for the Holocene baseline (11,700 BP—1850 ce). Blue denotes the area where the 15%–30% sea ice concentration isoband has persisted for ≥ 50% of Holocene summers. Figure S5. Three‐tiered [file ECE3-15-e71377-s001.docx]

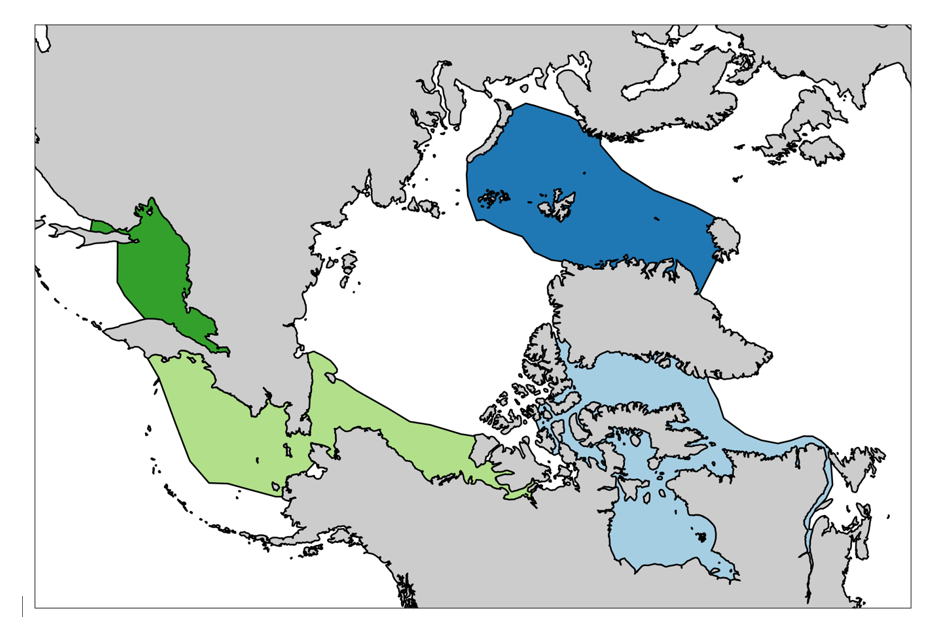


**SI Figure 1** – **Distribution of the four bowhead whale management stocks.** Whaling stocks are: East Greenland-Svalbard-Barents (dark blue); Canada-West Greenland (light blue); Bering-Chukchi- Beaufort (light green); Sea of Okhotsk (dark green).


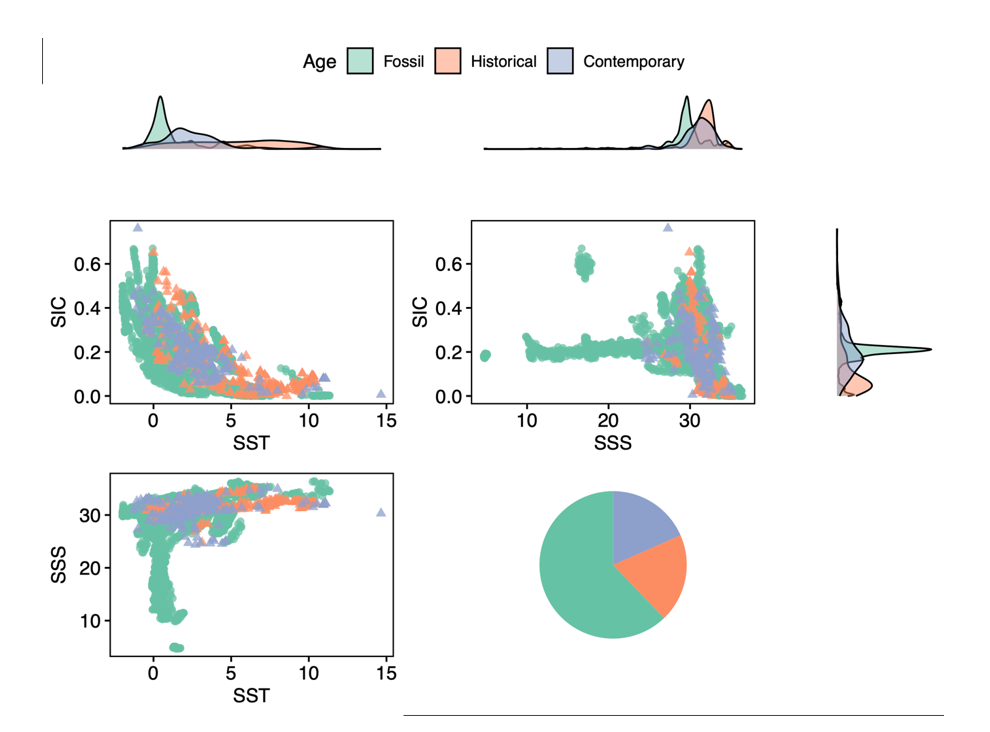


**SI Figure 2 – Pairwise 2-dimensional cross-sections of the bowhead whale hypervolume.** Colors represent post-processed occurrence records coming from fossil (green), historical (orange), and contemporary (blue) time periods. Density plots show the distribution of occurrence records along three of the four variables we included in the bowhead whale niche model: sea ice concentration (SIC); sea-surface temperature (SST); and sea-surface salinity (SSS). Bathymetry is not shown. The pie chart shows the proportion of records coming from each time period.


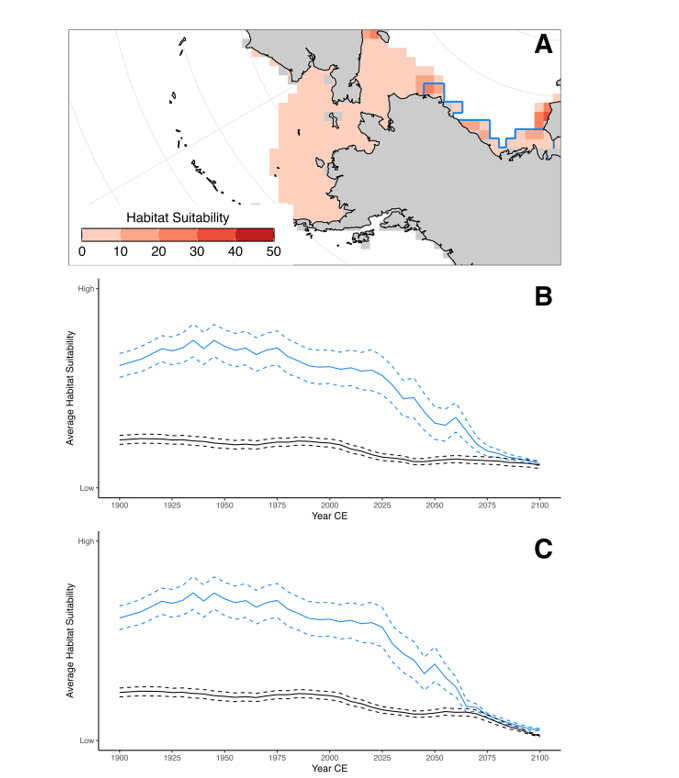


**SI Figure 3 – Relationship between habitat suitability and important current-day congregation sites for bowhead whales in the Bering-Chukchi-Beaufort stock**. **A –** summer-averaged (June-October; 1900-2020 CE) habitat suitability patterns, with summer high-congregation areas highlighted in blue along the Beaufort Sea coast. **B –** Habitat suitability patterns (1900-2100 CE) under a Shared Socioeconomic Pathway (SSP) 2-4.5 climate scenario in core use (blue) and non-core use areas (black). Dashed lines denote ± 1 standard error. **C –** Habitat suitability patterns (1900-2100 CE) under a SSP 5-8.5 climate scenario in core use (blue) and non-core use areas (black). Dashed lines denote ± 1 standard error. Our niche model positively identifies core congregation areas as having high habitat suitability. However, we project suitability in these core areas rapidly decline in the future regardless of SSP scenario, suggesting bowhead whales may cease being able to use them.


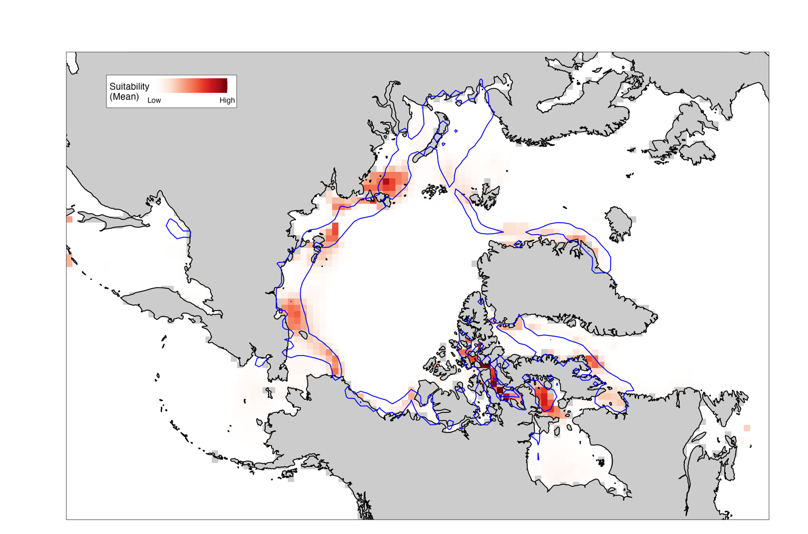


**SI Figure 4 – Spatial pattern of habitat suitability for the Holocene baseline (11,700 BP - 1850 CE).** Blue denotes the area where the 15-30% sea ice concentration isoband has persisted for ≥ 50% of Holocene summers.


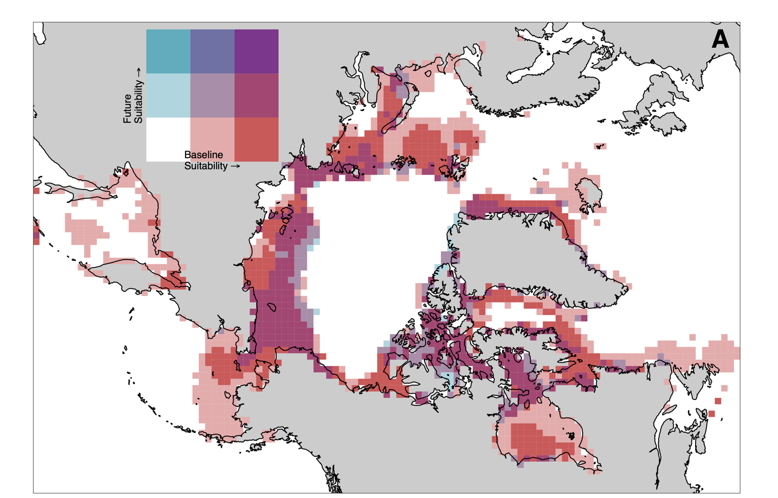

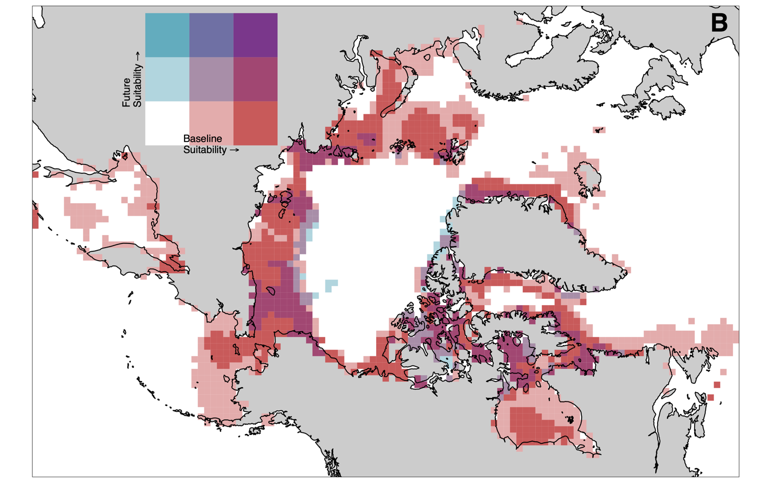


**SI Figure 5** – Three-tiered congruence maps (no suitability, low suitability, high suitability) between the Holocene baseline (11,700 BP – 1850 CE) and the two different Shared Socioeconomic Pathway (SSP) scenarios at 2100 CE (2-4.5 [**A**]; 5-8.5 [**B**]). Even in areas that will remain suitable by 2100 CE, suitability will remain low areas that still contain suitable habitat.


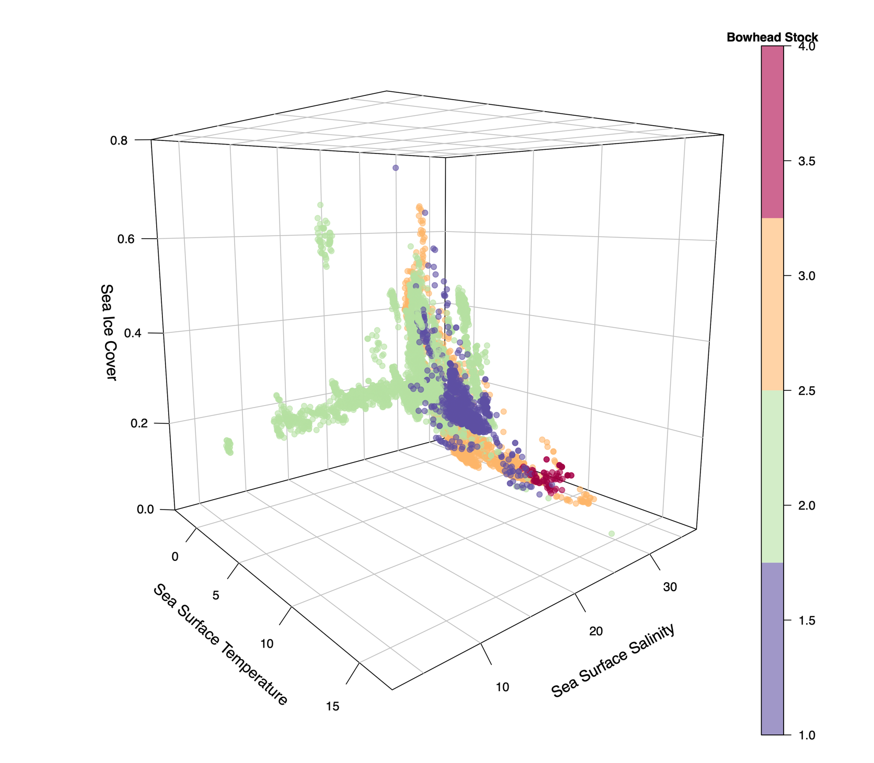


**SI Figure 6** – 3-dimensional representation of the hypervolume of climatic conditions that bowhead whales occupy based on fossil, historical, and contemporary occurrence data. Different colours show different bowhead stocks (East Greenland-Svalbard-Barents [blue]; 2 = Canada-West Greenland [green]; Bering-Chukchi-Beaufort [yellow]; 4 = Sea of Okhotsk [red]). Note that the occurrence relationship with bathymetry is not shown.


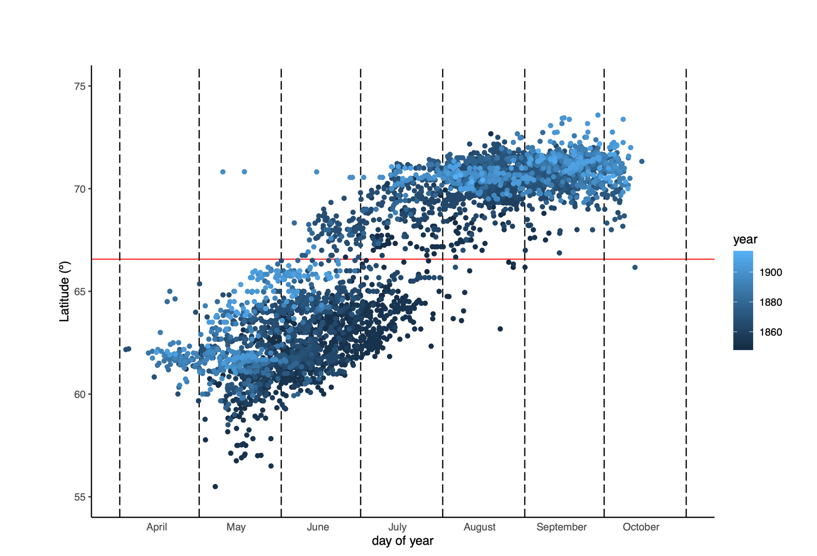


**SI Figure 7** – The latitude and day-of-year that historical Bering-Chukchi-Beaufort bowhead whale strikes were also observed on the same day as Arctic sea ice. The red line is an approximate latitude for the Bering Strait, indicating that bowhead whales were associated with summer sea ice south of the strait during the period of commercial harvest (1848-1914). Data are from Mahoney et al. (2011) based off original logbook extractions by Bockstoce and Botkin (1983).
