# Supplementary material for: 21st Century Sea Ice Loss Will Upend 11,700 Years of Stable Habitat for Bowhead Whales
Source: Ecol Evol. 2025 May 20;15(5):e71377. doi: 10.1002/ece3.71377 (PMC12092169; doi:10.1002/ece3.71377)
Supplement: Supplementary file 2 — Data S2. Table S1. Data sources and sample sizes of the bowhead whale occurrence data before and after data cleaning. For historical sources, citations marked with an asterisk were digitised in Smith et al. (2012). Some modern and historical data sources contain the same or redundant data, which were removed via spatial thinning (Aiello‐Lammens et al. 2015). The majority of modern occurrence records came from recent high‐resolution satellite telemetry data, which is why so much of it was removed via spatial thinning. Table S2. Percent declines in suitable habitable area for bowhead whales in 2100 ce under SSP 2–4.5 and SSP 5–8.5 emissions scenarios. Declines are shown for each population as well as declines across the entire bowhead whale circumpolar range. [file ECE3-15-e71377-s002.docx]

| Time period | Raw sample size | Cleaned sample size | Data Sources |
| --- | --- | --- | --- |
| Modern | 37,028 | 485 | (Chambault et al., 2022; de Korte and Belikov, 1994; GBIF, 2022; Ivashchenko and Clapham, 2010; Kovacs et al., 2020; Rogachev et al., 2008; Shpak et al., 2014; Wiig et al., 2010) |
| Historical | 3,374 |  | (Allen and Keay, 2004; Barkham, 1984; de Jong, 1972; Dijksman, 1986; Gray, 1931; Hacquebord et al., 2003; Hacquebord and Avango, 2009; Ivashchenko and Clapham, 2012; Jansen, 1865; Parry, 1824; Reeves, 1980*; Schokkenbroek, 2008; Smith et al., 2012; Townsend, 1935*; Vaughan, 1986) |
| Fossil | 823 | 823 | (Westbury et al., 2024) |
| Total | 41,225 | 1,308 |  |

**SI Table 1 – Data sources and sample sizes of the bowhead whale occurrence data before and after data cleaning**. For historical sources, citations marked with an asterisk were digitised in Smith et al. (2012). Some modern and historical data sources contain the same or redundant data, which were removed via spatial thinning (Aiello-Lammens et al., 2015). The majority of modern occurrence records came from recent high-resolution satellite telemetry data, which is why so much of it was removed via spatial thinning.

| **Bowhead whale population** | **SSP 2-4.5** | **SSP 5-8.5** |
| --- | --- | --- |
| East Greenland-Svalbard-Barents | 52.2% | 89.5% |
| Canada-West Greenland | 88.3% | 95.3% |
| Bering-Chukchi-Beaufort | 85.3% | 96.5% |
| Sea of Okhotsk | 100.0% | 100.0% |
| **Range-wide** | **63.9%** | **75.6%** |

**SI Table 2 – Percent declines in suitable habitable area for bowhead whales in 2100 CE under SSP 2-4.5 and SSP 5-8.5 emissions scenarios**. Declines are shown for each population as well as declines across the entire bowhead whale circumpolar range.

Citations

Aiello-Lammens, M.E., Boria, R.A., Radosavljevic, A., Vilela, B., Anderson, R.P., 2015. spThin: an R package for spatial thinning of species occurrence records for use in ecological niche models. Ecography 38, 541–545. https://doi.org/10.1111/ecog.01132

Allen, R.C., Keay, I., 2004. Saving the Whales: Lessons from the Extinction of the Eastern Arctic Bowhead. The Journal of Economic History 64, 400–432. https://doi.org/10.1017/S0022050704002748

Barkham, S.H., 1984. The Basque Whaling Establishments in Labrador 1536-1632 - A Summary. ARCTIC 37, 515–519. https://doi.org/10.14430/arctic2232

Chambault, P., Kovacs, K.M., Lydersen, C., Shpak, O., Teilmann, J., Albertsen, C.M., Heide-Jørgensen, M.P., 2022. Future seasonal changes in habitat for Arctic whales during predicted ocean warming. Science Advances 8, eabn2422. https://doi.org/10.1126/sciadv.abn2422

de Jong, C., 1972. Geschiedenis van de oude Nederlandse walvisvaart (Doctoral Thesis). [s.n.], [s.l.].

de Korte, J., Belikov, S.E., 1994. Observations of Greenland whales (Balaena mysticetus), Zemlya Frantsa-Iosifa. Polar Record 30, 135–136. https://doi.org/10.1017/S0032247400021367

Dijksman, R., 1986. Death on Jan Mayen: a whaling tragedy of 1634. Polar Record 23, 196–201. https://doi.org/10.1017/S0032247400028400

GBIF, 2022. GBIF Bowhead Whale Occurrence Download. https://doi.org/10.15468/dl.rvpdps

Gray, R.W., 1931. The Colour of the Greenland Sea and the Migrations of the Greenland Whale and Narwhal. The Geographical Journal 78, 284–290. https://doi.org/10.2307/1784901

Hacquebord, L., Avango, D., 2009. Settlements in an Arctic Resource Frontier Region. Arctic Anthropology 46, 25–39. https://doi.org/10.1353/arc.0.0028

Hacquebord, L., Steenhuisen, F., Waterbolk, H., 2003. English and Dutch Whaling Trade and Whaling Stations in Spitsbergen (Svalbard) before 1660. International Journal of Maritime History 15, 117–134. https://doi.org/10.1177/084387140301500207

Ivashchenko, Y., Clapham, P., 2012. Soviet catches of right whales Eubalaena japonica and bowhead whales Balaena mysticetus in the North Pacific Ocean and the Okhotsk Sea. Endang. Species. Res. 18, 201–217. https://doi.org/10.3354/esr00443

Ivashchenko, Y., Clapham, P., 2010. Bowhead whales Balaena mysticetus in the Okhotsk Sea. Mammal Review 40, 65–89. https://doi.org/10.1111/j.1365-2907.2009.00152.x

Jansen, M.H., 1865. Notes on the Ice between Greenland and Nova Zembla; being the results of investigations into the records of early Dutch voyages in the Spitzbergen Seas. Proceedings of the Royal Geographical Society of London 9, 163–181.

Kovacs, K.M., Lydersen, C., Vacquiè-Garcia, J., Shpak, O., Glazov, D., Heide-Jørgensen, M.P., 2020. The endangered Spitsbergen bowhead whales’ secrets revealed after hundreds of years in hiding. Biology Letters 16, 20200148. https://doi.org/10.1098/rsbl.2020.0148

Parry, W.E., 1824. Second Voyage for the Discovery of a North-West Passage from the Atlantic to the Pacific. Board of Longitude, London.

Reeves, R.R., 1980. Spitsbergen Bowhead Stock: A Short Review. Marine Fisheries Review 42, 65–69.

Rogachev, K.A., Carmack, E.C., Foreman, M.G.G., 2008. Bowhead whales feed on plankton concentrated by estuarine and tidal currents in Academy Bay, Sea of Okhotsk. Continental Shelf Research 28, 1811–1826. https://doi.org/10.1016/j.csr.2008.04.014

Schokkenbroek, J.C.A., 2008. Trying-out: an anatomy of Dutch whaling and sealing in the nineteenth century, 1815-1885. Aksant, Amsterdam.

Shpak, O.V., Meschersky, I.G., Chichkina, A.N., Kuznetsova, D.M., Paramonov, A.Yu., Rozhnov, V.V., 2014. New Data on the Okhotsk Sea Bowhead Whales (No. SC/65b/BRG17). International Whaling Comission.

Smith, T.D., Reeves, R.R., Josephson, E.A., Lund, J.N., 2012. Spatial and Seasonal Distribution of American Whaling and Whales in the Age of Sail. PLOS ONE 7, e34905. https://doi.org/10.1371/journal.pone.0034905

Townsend, C.H., 1935. The distribution of certain whales as shown by logbook records of American whaleships. Zoologica 19, 3–50.

Vaughan, R., 1986. Bowhead whaling in Davis Strait and Baffin Bay during the 18th and 19th Centuries. Polar Record 23, 289–299. https://doi.org/10.1017/S0032247400007117

Westbury, M.V., Brown, S.C., Cabrera, A.A., Morales, H.E., Ma, J., Rey-Iglesia, A., Dyke, A., Scharff-Olsen, C.H., Scott, M.B., Wiig, Ø., Bachmann, L., Kovacs, K.M., Lydersen, C., Ferguson, S.H., Racimo, F., Szpak, P., Fordham, D.A., Lorenzen, E.D., 2024. Four centuries of commercial whaling eroded 11,000 years of population stability in bowhead whales. https://doi.org/10.1101/2024.04.10.588858

Wiig, Ø., Bachmann, L., Øien, N., Kovacs, K.M., Lydersen, C., 2010. Observations of bowhead whales (Balaena mysticetus) in the Svalbard area 1940–2009. Polar Biol 33, 979–984. https://doi.org/10.1007/s00300-010-0776-1
